# Folliculin Regulates Osteoclastogenesis Through Metabolic Regulation

**DOI:** 10.1002/jbmr.3477

**Published:** 2018-06-26

**Authors:** Masaya Baba, Mitsuhiro Endoh, Wenjuan Ma, Hirofumi Toyama, Akiyoshi Hirayama, Keizo Nishikawa, Keiyo Takubo, Hiroyuki Hano, Hisashi Hasumi, Terumasa Umemoto, Michihiro Hashimoto, Nobuko Irie, Chiharu Esumi, Miho Kataoka, Naomi Nakagata, Tomoyoshi Soga, Masahiro Yao, Tomomi Kamba, Takashi Minami, Masaru Ishii, Toshio Suda

**Affiliations:** ^1^ International Research Center for Medical Sciences (IRCMS) Kumamoto University Kumamoto Japan; ^2^ Cancer Science Institute of Singapore National University of Singapore Centre for Translational Medicine Singapore; ^3^ Department of Cell Differentiation The Sakaguchi Laboratory of Developmental Biology School of Medicine Keio University Tokyo Japan; ^4^ Institute for Advanced Biosciences Keio University Yamagata Japan; ^5^ Immunology Frontier Research Center Osaka University Osaka Japan; ^6^ Department of Stem Cell Biology Research Institute National Center for Global Health and Medicine Tokyo Japan; ^7^ Department of Urology Yokohama City University Graduate School of Medicine Yokohama Japan; ^8^ Division of Reproductive Engineering Center for Animal Resources and Development (CARD) Kumamoto University Kumamoto Japan; ^9^ Department of Urology Faculty of Life Sciences Kumamoto University Kumamoto Japan; ^10^ Division of Molecular and Vascular Biology Institute of Resource Development and Analysis (IRDA) Kumamoto University Kumamoto Japan; ^11^ Department of Immunology and Cell Biology Graduate School of Medicine and Frontier Biosciences Osaka University Osaka Japan

**Keywords:** OSTEOCLAST, FOLLICULIN (FLCN), METABOLISM, OSTEOPOROSIS, TRANSCRIPTION FACTORS

## Abstract

Osteoclast differentiation is a dynamic differentiation process, which is accompanied by dramatic changes in metabolic status as well as in gene expression. Recent findings have revealed an essential connection between metabolic reprogramming and dynamic gene expression changes during osteoclast differentiation. However, the upstream regulatory mechanisms that drive these metabolic changes in osteoclastogenesis remain to be elucidated. Here, we demonstrate that induced deletion of a tumor suppressor gene, *Folliculin* (*Flcn*), in mouse osteoclast precursors causes severe osteoporosis in 3 weeks through excess osteoclastogenesis. *Flcn*‐deficient osteoclast precursors reveal cell autonomous accelerated osteoclastogenesis with increased sensitivity to receptor activator of NF‐κB ligand (RANKL). We demonstrate that Flcn regulates oxidative phosphorylation and purine metabolism through suppression of nuclear localization of the transcription factor Tfe3, thereby inhibiting expression of its target gene *Pgc1*. Metabolome studies revealed that *Flcn*‐deficient osteoclast precursors exhibit significant augmentation of oxidative phosphorylation and nucleotide production, resulting in an enhanced purinergic signaling loop that is composed of controlled ATP release and autocrine/paracrine purinergic receptor stimulation. Inhibition of this purinergic signaling loop efficiently blocks accelerated osteoclastogenesis in *Flcn*‐deficient osteoclast precursors. Here, we demonstrate an essential and novel role of the Flcn‐Tfe3‐Pgc1 axis in osteoclastogenesis through the metabolic reprogramming of oxidative phosphorylation and purine metabolism. © 2018 The Authors *Journal of Bone and Mineral Research* published by Wiley Periodicals, Inc. on behalf of American Society for Bone and Mineral Research (ASBMR).

## Introduction

Cellular metabolism regulates cell proliferation and differentiation. Recent emerging developments in the research field of metabolism are defining many novel molecular mechanisms involved in cell differentiation. Osteoclastogenesis is one of the most dramatic cell differentiation events, which is accompanied by dynamic changes in cellular metabolism and gene expression. Thus, the osteoclast differentiation process is one of the best physiological conditions in which to investigate novel mechanisms of metabolic regulation that drive cell differentiation. It is known that glucose metabolism shifts toward a more oxidative state when osteoclast precursors differentiate to pre‐osteoclasts and osteoclasts upon receptor activator of NF‐κB ligand (RANKL) stimulation. Induction of *Pgc1β* expression by RANKL stimulation causes the aforementioned metabolic reprogramming of osteoclast precursors, resulting in a dramatic change in cellular metabolite profiling, which in turn regulates gene expression that facilitates osteoclastogenesis.^1^, ^2^, ^3^

*Folliculin* (*FLCN*) was originally identified as a tumor suppressor gene that is responsible for a hereditary kidney cancer syndrome, Birt‐Hogg‐Dubé (BHD) syndrome. Folliculin (FLCN) was a novel protein without any known functional domain when it was first identified in 2002.^4^ Subsequent research mainly in *Flcn*‐deficient mouse models has uncovered multiple functions of Flcn in metabolic regulation and a fundamental role in many physiological processes in vivo.^5^, ^6^, ^7^, ^8^, ^9^, ^10^, ^11^, ^12^, ^13^, ^14^ We have previously shown that Flcn regulates oxidative metabolism through the expression control of *Pgc1*α in murine skeletal muscle and cardiomyocytes. Loss of *Flcn* causes increased mitochondrial biogenesis, resulting in elevated levels of cellular ATP.^15^, ^16^

Here, we found that bone marrow targeted *Flcn* KO mice showed a severe osteoporotic phenotype with increased osteoclast number and bone absorption. We hypothesized that Flcn might have an essential role in osteoclast differentiation through metabolic regulation and aimed to clarify the role of FLCN in osteoclastogenesis from the aspect of metabolism. We found that *Flcn* deficiency enhanced a metabolic shift toward oxidative phosphorylation and increased nucleotide production, which resulted in a dramatic elevation of purinergic metabolites in *Flcn*‐deficient osteoclast precursors. Purinergic metabolites themselves can stimulate purinergic receptors, thereby establishing a purinergic signaling loop. Moreover, we showed that the intervention of a purinergic signaling loop could block the accerelated osteoclastogenesis of *Flcn*‐deficient cells. Our novel findings will provide new insights into the metabolic regulation of osteoclast differentiation by Flcn and the basis for understanding the role and upstream regulation mechanism of metabolic reprogramming and purinergic signaling in osteoclastogenesis.

## Materials and Methods

### Mice and bone analysis

*Flcn* conditional knockout mice were generated as previously described.^5^ An *Mx1‐Cre* 17 transgene was introduced into *Flcn^f/f^* mice. *Flcn^f/f^;Mx1‐Cre+* mice and *Flcn^f/f^* littermates mice were injected intraperitoneally at 11 weeks of age with 300 μg of polyinosinic–polycytidylic acid solution (pIpC) (tlrl‐pic, Invivogen) 2 times every other day. Three‐dimensional microcomputed tomography (µCT) analyses were performed as described previously.^2^ Bone morphometric analyses were performed by KUREHA Special Laboratory. The nomenclature, symbol, and units of bone histomorphometry and bone morphometry were used according to Bouxsein and colleagues and Dempster and colleagues.^18^, ^19^ All animal experiments were approved by Kumamoto University Animal Care and Use Committee and performed in accordance with the legal requirements of the Association for Assessment and Accreditation of the Laboratory Animal Care International and the guidelines of Kumamoto University for Animal Care and Use Committee. All mice were housed in an accredited animal facility of Kumamoto University under a 12‐hour light/dark cycle with access to regular food and water ad libitum.

### Cell culture

Raw264.7 cells were cultured with RPMI‐1640 supplemented with 10% FCS (HyClone, GE Healthcare, Piscataway, NJ, USA), 100 U/mL penicillin, 100 µg/mL streptomycin. Raw 264.7 cells were transfected with the expression construct (pCAG‐Tfe3.GR‐IRES‐Puro) by using Lipofectamine 2000 (Thermo Fisher Scientific, Waltham, MA, USA), followed by clonal selection with 3.0 µg/mL of puromycin. Raw 264.7 cell clones stably expressing a scrambled or a *Flcn*‐specific shRNA were established by utilizing lentiviruses produced by the modified pLVX‐shRNA2‐ZsGreen system. The single clones of ZsGreen‐positive cells were isolated by FACSAriaII (Becton Dickinson, Franklin Lakes, NJ, USA). The shRNA for mouse *Flcn* (target sequence: CTTCAAGTCTCTTCGACACAT) was selected according to a previous report.^20^ For siRNA‐mediated gene knockdown, 30 pmol of siRNA was transfected using Screen Fect siRNA (Wako, Richmond, VA, USA) into 2 × 10^5^ cells per well in a 12‐well plate. To collect conditioned culture media, 450 pmol of siRNA was transfected into 3 × 10^6^ cells per 10 cm culture dish. The following siRNAs were utilized: Flcn: Stealth siRNA for *Flcn*, MSS278133; UUCAAACGCUGAAUGGACCAGGUUC (Life Technologies, Carlsbad, CA, USA) and Stealth siRNA negative control med GC, 12935‐300 (Life Technologies). For double knockdown experiments, the Flcn‐targeting shRNA lentivirus vector with a Puromycin‐resistance gene (pLV[shRNA]‐Puro‐U6>mFlcn[shRNA#6]) and the Tfe3‐targeting shRNA lentivirus vector with a Blasticidin‐resistance gene (pLV[shRNA]‐Bsd‐U6>mTfe3[shRNA#11]) were purchased from VectorBuilder (Santa Clara, CA, USA). Chemicals used for cell culture experiments were as follows: CV1808 (#1710, Tocris Bioscience, Bristol, UK), FCCP (#C2920, Sigma, St. Louis, MO, USA), oATP (#A6779, Sigma), Mefloquin (#SC‐211784, Santa Cruz Biotechnology, Dallas, TX, USA), and ADA (#52544, Sigma).

### In vitro osteoclast differentiation assay

An amount of 1.5 × 10^4^ Raw264.7 cells/well in a 24‐well plate were transfected with scramble siRNA or *Flcn*‐targeted siRNA. After 24 hours, the medium was changed to differentiation medium: MEMα containing 10% FCS (HyClone) without or with 40 ng/mL of RANKL (Oriental Yeast, Tokyo, Japan). Medium was changed every other day. Then, cells were fixed and subjected to TRAP staining using Acid Phosphatase, Leukocyte (TRAP) Kit (Sigma‐Aldrich). TRAP‐positive multinucleated (3 or more nuclei) and large (diameter ≥ 200 μm) cells were counted as osteoclasts. Images of each well were obtained by BZ‐X700 microscope (Keyence, Itasca, IL, USA). Areas of TRAP‐positive multinucleated osteoclasts were measured by BZ‐X Analyzer software (Keyence).

### Tetramethylrhodamine, ethyl ester (TMRE)‐mitochondrial membrane potential assay by flow cytometry

BM mononuclear cells were treated with tetramethylrhodamine, ethyl ester (TMRE) (Enzo, Farmingdale, NY, USA) at a final concentration of 25 μM for 30 minutes at 37°. Subsequently, the cells were washed, incubated with Fc block (BD Biosciences, San Jose, CA, USA) and stained with anti‐Mac‐1 (M1/70, BD Biosciences) on ice and subjected to flow cytometry analysis.

### cAMP measurement

RAW264.7 cells were transfected with scramble siRNA or *Flcn*‐targeted siRNA. After 24 hours, the medium was changed to differentiation medium with RANKL, followed by 48‐hour culture and harvest for cAMP measurement. Intracellular cAMP was measured by competitive enzyme immunoassay (EIA) according to the manufacturer's instructions (Cayman Chemical, Ann Arbor, MI, USA).

### Quantitative RT‐PCR (qRT‐PCR)

Total RNA was isolated using TRIzol Reagent (Thermo Fisher Scientific) and reverse transcribed to cDNA using ReverTra Ace qPCR RT Master Mix (Toyobo, Osaka, Japan). qPCR assays were performed with the Roche Lightcycler 96 system (Roche, Mannheim, Germany) and the Thunderbird Sybr qPCR Mix (Toyobo). Primer sequences are given in Supplemental Table S1. Each value was normalized using the value of *Rps18* as an internal control.

### DNA microarray analysis

Raw264.7 cells were transfected with scramble or *Flcn* targeting siRNA, followed by a medium change at 24 hours after transfection. Cells were cultured after an additional 48 hours and total RNA was collected and purified using the RNeasy Micro Kit (Qiagen, Hilden, Germany). cDNA preparation and hybridization of the probe arrays were performed according to the manufacturer's instructions (Affymetrix, Santa Clara, CA, USA). Affymetrix GeneChip Mouse Genome 430 2.0 arrays were applied. Data were processed using the Affymetrix GeneChip Operating Software (GCOS) Version 1.0. Data are available at the NCBI GEO database under accession number GSE115084.

### Immunocytochemistry (ICC)

Tfe3 staining with anti TFE3 antibody (Sigma, #HPA023881) was performed as previously described.^21^ Fluorescence images were obtained using a confocal laser‐scanning microscope (Nikon, A1R). Scanning was performed in sequential laser emission mode to avoid scanning at other wavelengths.

### Chromatin immunoprecipitation (ChIP)‐qPCR

RAW264.7 cells expressing Tfe3‐GR were cultured for 48 hours with or without 100 nM of Dexamethasone. SimpleChIP Plus Enzymatic Chromatin IP Kit (Magnetic Beads) (#9005, Cell Signaling, Danvers, MA, USA) was utilized for ChIP. Cell cross‐linking, chromatin preparation, and chromatin immunoprecipitation by anti TFE3 antibody (Sigma, #HPA023881) was performed according to the manufacturer's instructions. Primer sequences for qPCR assays are given in Supplemental Table S2.

### Metabolome analysis

Raw264.7 cells were transfected with scramble or *Flcn*‐targeted siRNA, followed by a medium change to MEMα containing 10% FCS (HyClone) and 40 ng/mL of RANKL at 24 hours after transfection. After 48‐hour culture, the cells were washed twice with 5% (w/v) aqueous mannitol solution. Then, 1 mL of methanol containing the internal standards (25 µmol/L each of methionine sulfone and D‐camphor‐10‐sulfonic acid) was added. After 10‐minute incubation, the 400 µL of dissolved solution was mixed with 200 μL of Milli‐Q water and 400 μL of chloroform. After centrifugation, the separated methanol‐water layer was ultrafiltered using a 5‐kDa cut‐off filter (Human Metabolome Technologies, Tsuruoka, Japan) to remove proteins. The filtrate was dried using an vacuum centrifuge and dissolved in 50 µL of Milli‐Q water containing 200 µmol/L reference compounds (3‐aminopyrrolidine and trimesic acid) before CE‐MS analysis. CE‐MS‐based metabolomic profiling and data analysis were performed essentially as described.^22^, ^23^, ^24^, ^25^, ^26^

### Statistical analysis

Experimental data are summarized as the mean values with standard deviation SD. Statistical analyses were performed using a two‐tailed unpaired *t* test with or without Welch's correction. For multiple comparisons, one‐way ANOVA Dunnett's multiple comparisons test was utilized (GraphPad Prism 6, GraphPad, La Jolla, CA, USA). Differences were considered to be statistically significant at a value of *p *< 0.05.

## Results

### Severe osteoporosis in bone marrow targeted *Flcn* knockout mice caused by enhanced osteoclastogenesis

**Figure 1:**
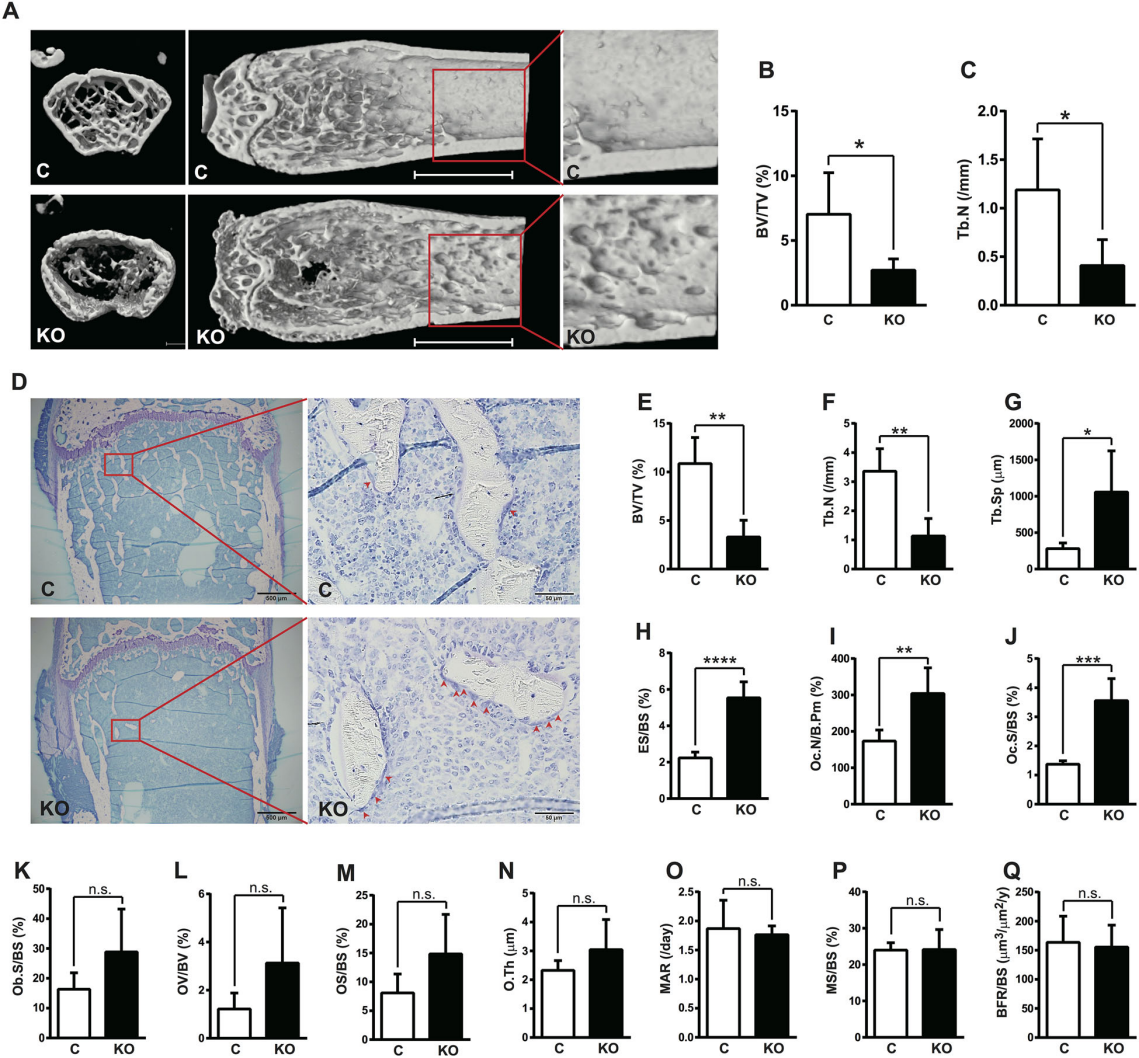
Severe osteoporosis with increased osteoclast number and accelerated bone absorption in bone marrow targeted *Flcn* KO mice. (*A*) Representative images showing μCT of femora from *Flcn ^f/f^;Mx1‐Cre−* (C: *Flcn* WT) mice (upper panel) and *Flcn ^f/f^;Mx1‐Cre^+^*(KO: *Flcn* KO) mice (lower panel). Mice were treated with pIpC at 11 weeks of age and dissected 3 weeks after pIpC treatment. *Flcn* KO mice demonstrate dramatically reduced trabecular bone and thinner cortical bone with many abnormal dents. Scale bars = 2 mm. (*B*, *C*) Bone morphological parameters calculated from μCT scans demonstrate severe osteoporosis in *Flcn* KO mice. Data are presented as mean with SD (*n* = 5 mice for each group, unpaired *t* test: *p < 0.05). Bone volume fraction (BV/TV) and trabecular number (Tb.N) were significantly lower in *Flcn* KO mice. (*D*) Representative images of the Toluidine blue staining of femora from *Flcn* WT (C) (*n* = 5) and *Flcn* KO (KO) (*n* = 4) mice at 3 weeks after pIpC injection. Osteoclasts are indicated by arrowheads. Scale bars = 500 μm (left panel), 50 μm (right panel). (*E–Q*) Histomorphometric analysis on Toluidine blue–stained femora demonstrates osteoporosis and accelerated bone absorption. Data are presented as mean with SD (*n* = 5 for *Flcn* WT; *n* = 4 for *Flcn* KO, unpaired *t* test: n.s. = not significant, *p < 0.05, **p < 0.01, ***p < 0.001, ****p < 0.0001). (*E–G*) Bone volume (BV/TV), trabecular number (Tb.N), and trabecular separation (Tb.Sp) were significantly lower in *Flcn* KO mice. (*H*, *I*) Eroded surface (ES/BS) and osteoclast number (Oc.N/B.Pm) were significantly higher in *Flcn* KO mice. (*J*) Osteoclast surface (Oc.S/BS) was significantly higher in *Flcn* KO mice. (*K–N*) Osteoblast surface (Ob.S/BS), osteoid volume (OV/BV), and osteoid surface (OS/BS) did not show statistical significance between *Flcn* WT and *Flcn* KO mice. (*O–Q*) Dynamic bone histomorphometric analysis with calcein labeling demonstrated no significant difference in dynamic bone formation parameters, including mineral apposition rate (MAR), mineralizing surface (MS/BS), and bone formation rate (BFR/BS) between *Flcn* WT and *Flcn* KO mice.

To investigate the significance of metabolic reprogramming in osteoclast differentiation, we conditionally deleted *Flcn* by utilizing *Mx1* promoter‐driven *Cre* transgenic mice.^17^ *Flcn* knockout mice (*Flcnf/f, Mx1‐cre*) demonstrated severe osteoporosis with acute progression in 3 weeks after *Flcn* deletion induced by pIpC injection. 3D reconstruction of femora by micro‐CT analysis revealed severe osteoporosis of *Flcn* knockout mice. As shown in Fig. 1 *A*, trabecular bone in *Flcn* knockout mice was dramatically reduced. Furthermore, the inner surface of the cortical bone in *Flcn* knockout mice was irregular with many dents or holes (Fig. 1 *A*). Quantified bone volume fraction and trabecular numbers in *Flcn* knockout mice were reduced more than 50% compared with control *Flcnf/f* mice (Fig. 1 *B*, *C*). Bone histomorphometric analysis also revealed severe osteoporosis in *Flcn* knockout mice with reduced bone volume, trabecular number, and increased trabecular separation (Fig. 1 *D–G*). The eroded surface generated by osteoclasts was significantly greater in *Flcn* knockout mice (Fig. 1 *H*). In support of these data, osteoclast number (Fig. 1 *I*) and osteoclast surface (Fig. 1 *J*) were significantly greater in *Flcn* knockout mice compared with controls. These observations of static bone resorption parameters strongly support enhanced osteoclastogenesis in *Flcn* knockout mice. On the other hand, there were no significant differences in static bone formation parameters, including osteoblast surface, osteoid volume, osteoid surface, and osteoid thickness (Fig. 1 *K–N*). Dynamic bone formation parameters including mineral apposition rate, mineralizing surface, and bone formation rate demonstrated no statistically significant differences between *Flcn* knockout mice and control mice (Fig. 1 *O–Q*). These data demonstrate that severe osteoporosis in *Flcn* knockout mice is caused by enhanced osteoclastogenesis.

### Cell autonomous enhancement of osteoclastogenesis in *Flcn*‐deficient osteoclast precursors

**Figure 2:**
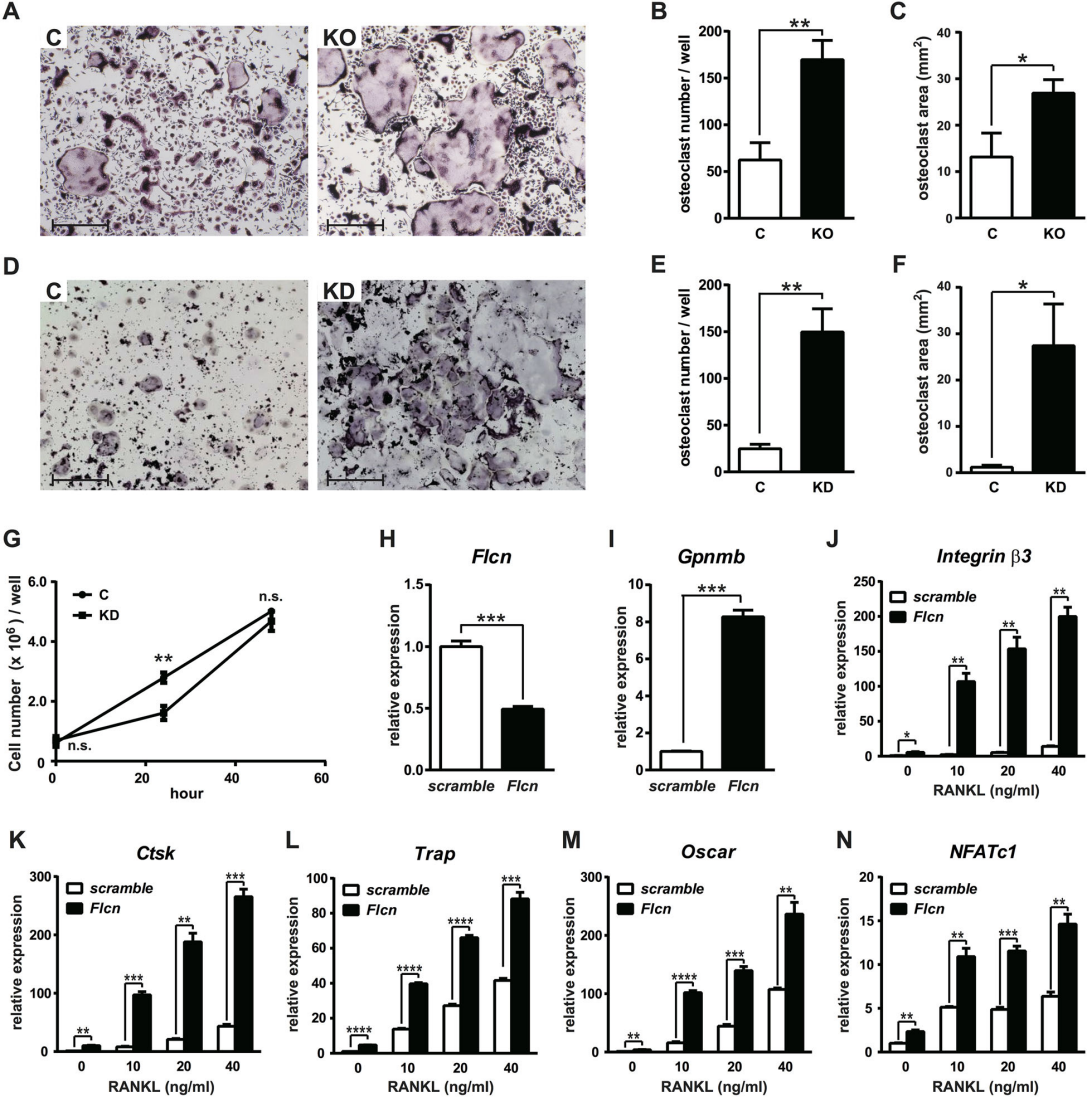
Enhanced osteoclastogenesis in *Flcn*‐deficient osteoclast precursor cells. (*A*) Osteoclast precursors from *Flcn ^f/f^;Mx1‐Cre*− (C) mice and *Flcn ^f/f^;Mx1‐Cre^+^*(KO) mice were cultured with M‐CSF (100 ng/mL) and RANKL (100 ng/mL) for 96 hours, followed by TRAP staining. More multinucleated TRAP‐positive osteoclasts were differentiated from *Flcn*‐deficient osteoclast precursor cells. Scale bars = 400 μm. (*B*) The number of multinucleated (3 or more nuclei) TRAP‐positive osteoclasts in 24‐well plates. (*C*) The area of multinucleated TRAP‐positive osteoclasts in 24‐well plates. There were significantly more osteoclasts differentiated from *Flcn*‐deficient osteoclast precursors. Data represent means ± SD (triplicate, unpaired *t* test: *p < 0.05, **p < 0.01). (*D*) Mouse osteoclast precursor cell line RAW264.7 cells were transfected with scramble siRNA (*C*) or *Flcn* siRNA (KD). At 24 hours after transfection, culture medium was changed to MEMα containing 40 ng/mL of RANKL and cultured for additional 120 hours for osteoclastic differentiation, followed by TRAP staining. Scale bars = 1 mm (*E*) The number of multinucleated (3 or more nuclei) TRAP‐positive osteoclasts in 24‐well plates. (*F*) The area of multinucleated TRAP‐positive osteoclasts in 24‐well plates. Data represent means ± SD (triplicate, unpaired *t* test: *p < 0.05, **p < 0.01). (*G*) RAW264.7 cells were transfected with scramble siRNA (C) or *Flcn* siRNA (KD) in 6‐well plates. At 24 hours after transfection, culture medium was changed to MEMα containing 40 ng/mL of RANKL and the number of Trypan blue–negative viable cells were counted at 0 hour, 24 hours, and 48 hours after the medium change. Data represent means ± SD (triplicate, unpaired *t* test: **p < 0.01). (*H*) *Flcn* knockdown was confirmed by qRT‐PCR on siRNA transfected RAW264.7 cells. (*I*) Elevated *Gpnmb* expression, a readout for Flcn deficiency, was observed in *Flcn* knockdown RAW264.7 cells. (*J–N*) RAW264.7 cells were transfected with scramble siRNA or *Flcn* siRNA. At 24 hours after transfection, culture medium was changed to differentiation medium containing different concentrations of RANKL. After additional 48 hours of culture with RANKL/MEMα, mRNA expression was measured by qRT‐PCR to evaluate osteoclastic differentiation. Readout genes for osteoclastogenesis including *Integrin β3* (*J*), *Cathepsin K* (*K*), *Trap* (*L*), and *Oscar* (*M*) were expressed at significantly higher levels in *Flcn* knockdown cells. (*N*) The expression of *Nfatc1* was elevated in *Flcn* knockdown cells even in the absence of RANKL stimulation. Data represent means ± SD (triplicate, unpaired *t* test: *p < 0.05, **p < 0.01, ***p < 0.001, ****p < 0.0001). Representative data from at least three independent experiments are shown.

To investigate if enhancement of osteoclastogenesis in *Flcn* knockout mice is caused by a cell autonomous mechanism or microenvironment‐dependent mechanism, we performed an in vitro osteoclast differentiation assay. Osteoclast precursors from bone marrow of *Flcn* knockout mice and control mice were cultured with M‐CSF and RANKL for 4 days and stained for TRAP activity. TRAP‐positive multinucleated osteoclast numbers and areas were quantified. *Flcn* knockout osteoclast precursors have greater differentiation ability than wild‐type control cells (Fig. 2 *A–C*). The cell autonomous role of Flcn in osteoclastogenesis was also evaluated in the RAW264.7 osteoclast precursor cell line. siRNA mediated *Flcn* knockdown in RAW264.7 cells dramatically enhanced osteoclastogenesis with a low dose of RANKL (40 ng/mL) in 120 hours (Fig. 2 *D–F*). *Flcn* knockdown RAW264.7 cells did not proliferate faster than control cells, which indicates that accelerated osteoclastogenesis of *Flcn*‐deficient osteoclast precursors is not caused by increased cell proliferation (Fig. 2 *G*). *Flcn* knockdown efficacy was confirmed by quantifying gene expression of *Gpnmb*, whose elevation is a specific readout for *Flcn* deficiency^21^ (Fig. 2 *H*, *I*). Expression of readout genes for osteoclast differentiation, including *Integrin β3*, *Cathepsin K*, *Trap*, and *Oscar*, was measured by qRT‐PCR in RAW264.7 cells transfected with *Flcn*‐targeted siRNA or scramble siRNA (Fig. 2 *J–M*). *Flcn* knockdown made RAW264.7 cells sensitive to RANKL stimulation, thereby promoting osteoclast differentiation, which was demonstrated by greater induction of readout gene expression. Nfatc1 is known as a master transcription factor for osteoclastogenesis and its expression is induced upon RANKL stimulation.^27^ Intriguingly, *Nfatc1* expression was increased in *Flcn* knockdown cells even without RANKL stimulation and was dramatically elevated upon RANKL stimulation for 48 hours (Fig. 2 *N*). Taken together, these data demonstrate the cell autonomous enhancement of osteoclastogenesis by loss of *Flcn* in osteoclast precursors.

### Involvement of Flcn‐Tfe3‐Pgc1 axis in osteoclastogenesis

**Figure 3:**
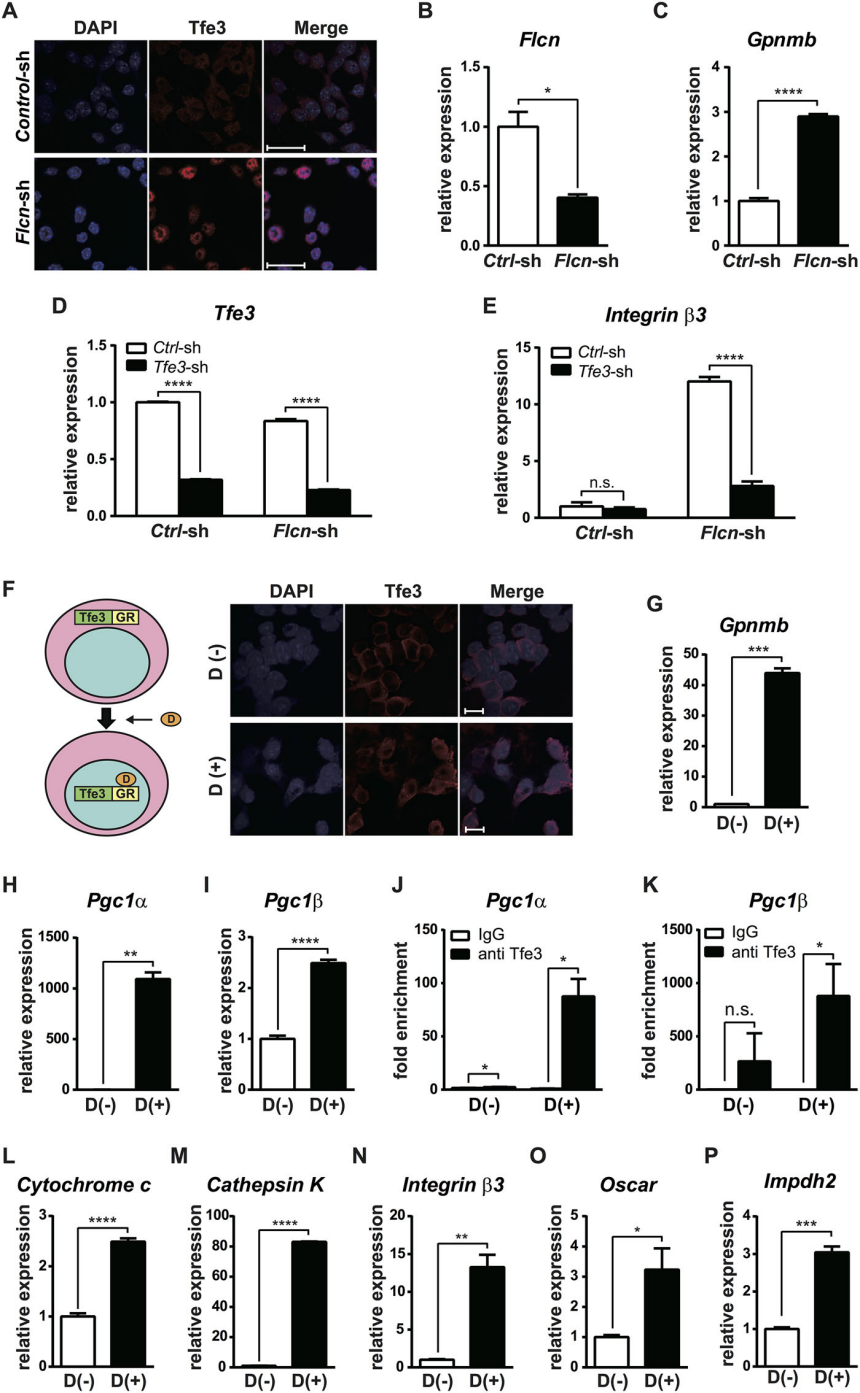
Flcn regulates osteoclastogenesis partly through Tfe3‐Pgc1 axis. (*A*) Immunofluorescent staining of endogenous Tfe3 demonstrated nuclear localization of Tfe3 in shRNA mediated *Flcn* knockdown RAW264.7 cells, whereas control RAW264.7 cells revealed cytoplasmic localization of Tfe3. Blue: DAPI; red: Tfe3. Scale bars = 20 μm. (*B*, *C*) qRT‐PCR on RAW264.7 cell lines stably transfected with *control*‐shRNA vector and *Flcn*‐shRNA vector. Efficient *Flcn* knockdown was confirmed by lower *Flcn* mRNA expression (*B*) and elevated *Gpnmb* mRNA expression (*C*) by qRT‐PCR. (*D*) Flcn targeted or control shRNA encoding lentiviral vectors with puromycin resistance cassette were co‐infected with Tfe3 targeted or control shRNA encoding lentiviral vectors with Blasticidin resistance cassette into RAW264.7 cells in a 12‐well plate. At 24 hours after infection, medium was changed to selection medium containing 10 μg/mL of Puromycin and 6 μg/mL of Blasticidin. After the 48‐hour culture with selection medium, cells were reseeded with MEMα medium and cultured for an additional 72 hours, followed by harvest and qRT‐PCR. Efficient shRNA‐mediated *Tfe3* knockdown was confirmed by qRT‐PCR. (*E*) *Integrin β3* gene expression was quantified on the same samples in Fig. 3 *D*. Additional *Tfe3* knockdown on *Flcn* knockdown RAW264.7 cells significantly suppressed *Integrin β3* expression. (*F*) Immunofluorescent staining with anti‐TFE3 antibody on RAW264.7 cells expressing Tfe3‐GR (Tfe3‐GR#1), which were cultured without dexamethasone or with 100 nM of dexamethasone. Dexamethasone‐dependent forced nuclear translocation of Tfe3‐GR was observed. Blue: DAPI, red: Tfe3. Scale bars = 10 μm. (*G–L*) Tfe3‐GR cells were cultured for 48 hours with or without dexamethasone, followed by gene expression quantification by qRT‐PCR. (*G*) *Gpnmb*, a known transcriptional target gene of Tfe3, was significantly elevated by forced nuclear translocation of Tfe3. (*H*, *I*) *Pgc1α* and *Pgc1β* master transcriptional regulators of mitochondrial biogenesis were significantly elevated by Tfe3 nuclear localization. (*J*, *K*) Tfe3‐GR#1 cells were cultured with or without 100 nM of dexamethasone, followed by chromatin immunoprecipitation (ChIP) with control IgG and anti‐TFE3 antibody. Tfe3 binding to M‐box consensus sequences in the *Pgc1α* gene (*J*) and the *Pgc1β* gene (*K*) were quantified by qPCR on ChIP samples and the fold enrichments relative to the input chromatins were calculated. (*L*) The expression of Cytochrome c, a target gene of Pgc1, was quantified by qRT‐PCR. (*M–O*) qRT‐PCR results indicate that the expression of readout genes for osteoclast differentiation was dramatically induced by forced nuclear translocation of Tfe3 even without RANKL stimulation. (*P*) qRT‐PCR of *Impdh2*, which is a known target of MITF. Data represent means ± SD (triplicate, unpaired *t* test: *p < 0.05, **p < 0.01, ***p < 0.001, ****p < 0.0001). Representative data from at least three independent experiments are shown.

We and others have previously shown that FLCN negatively regulates transcriptional activity of TFE3 transcription factor by regulating its nuclear localization.^20^, ^21^, ^28^ We confirmed that Tfe3 localized predominantly in the nucleus of *Flcn*‐deficient RAW264.7 cells, whereas Tfe3 localized in the cytoplasm of control cells (Fig. 3 *A*). Increased transcriptional activity of Tfe3 in *Flcn* knockdown cells was confirmed by elevated *Gpnmb* expression, which is a known Tfe3 transcriptional target gene (Fig. 3 *C*). To see the contribution of Tfe3 activation for enhanced osteoclastogenesis in *Flcn*‐deficient osteoclast precursors, we performed shRNA‐mediated knockdown of *Tfe3* in *Flcn* knockdown RAW264.7 cells (Fig. 3 *D*). As shown in Fig. 3 *E*, increased *Integrin β3* expression by *Flcn* knockdown was greatly suppressed by additional *Tfe3* knockdown. To see the importance of Tfe3 nuclear localization for enhanced osteoclastogenesis in *Flcn*‐deficient osteoclast precursors, we have generated a RAW264.7 cell line stably expressing Tfe3‐GR fusion protein. Tfe3‐GR is an artificial fusion protein, in which glucocorticoid receptor ligand binding domain (GR) is fused to Tfe3. Thus, Tfe3‐GR nuclear translocation can be induced in *Flcn* intact cells by addition of dexamethasone to the culture medium (Fig. 3 *F*). The dramatic induction of Gpnmb expression by dexamethasone treatment confirmed an efficient activation of Tfe3 (Fig. 3 *G*). Pgc1β is known to have an essential role in osteoclastogenesis.^1^ We observed a significant increase in *Pgc1β* expression caused by forced nuclear localization of Tfe3‐GR, suggesting possible transcriptional regulation of *Pgc1β* by Tfe3, as has been demonstrated for *Pgc1α* (Fig. 3 *L*, *M*).^29^ Then, we confirmed the direct binding of Tfe3 to the M‐box motifs in *Pgc1β* gene as well as *Pgc1α* gene by ChIP‐qPCR, which was specifically observed in Tfe3 activated cells (Fig. 3 *J*, *K*). Indeed, the expression of *Cytochrome c*, a target gene of Pgc1, was induced upon Tfe3 activation (Fig. 3 *L*). In addition, readout genes for osteoclast differentiation, *Integrin β3*, *Cathepsin K*, and *Oscar*, were significantly induced by forced nuclear localization of Tfe3 in *Flcn* intact cells even without RANKL stimulation (Fig. 3 *M–O*). Previously, we have shown an essential role of Impdh2, the rate‐limiting enzyme for purine metabolism, whose expression is regulated by MITF transcription factor, for HSC proliferation.^30^ Activation of Tfe3, an MITF family transcription factor, also induced Impdh2 expression significantly (Fig. 3 *P*). These data demonstrate that the nuclear translocation of Tfe3 induced by Flcn loss contributes to the enhancement of osteoclastogenesis presumably through the activation of its target genes, including *Pgc1*.

### Flcn regulates mitochondrial biogenesis and purine metabolism

**Figure 4:**
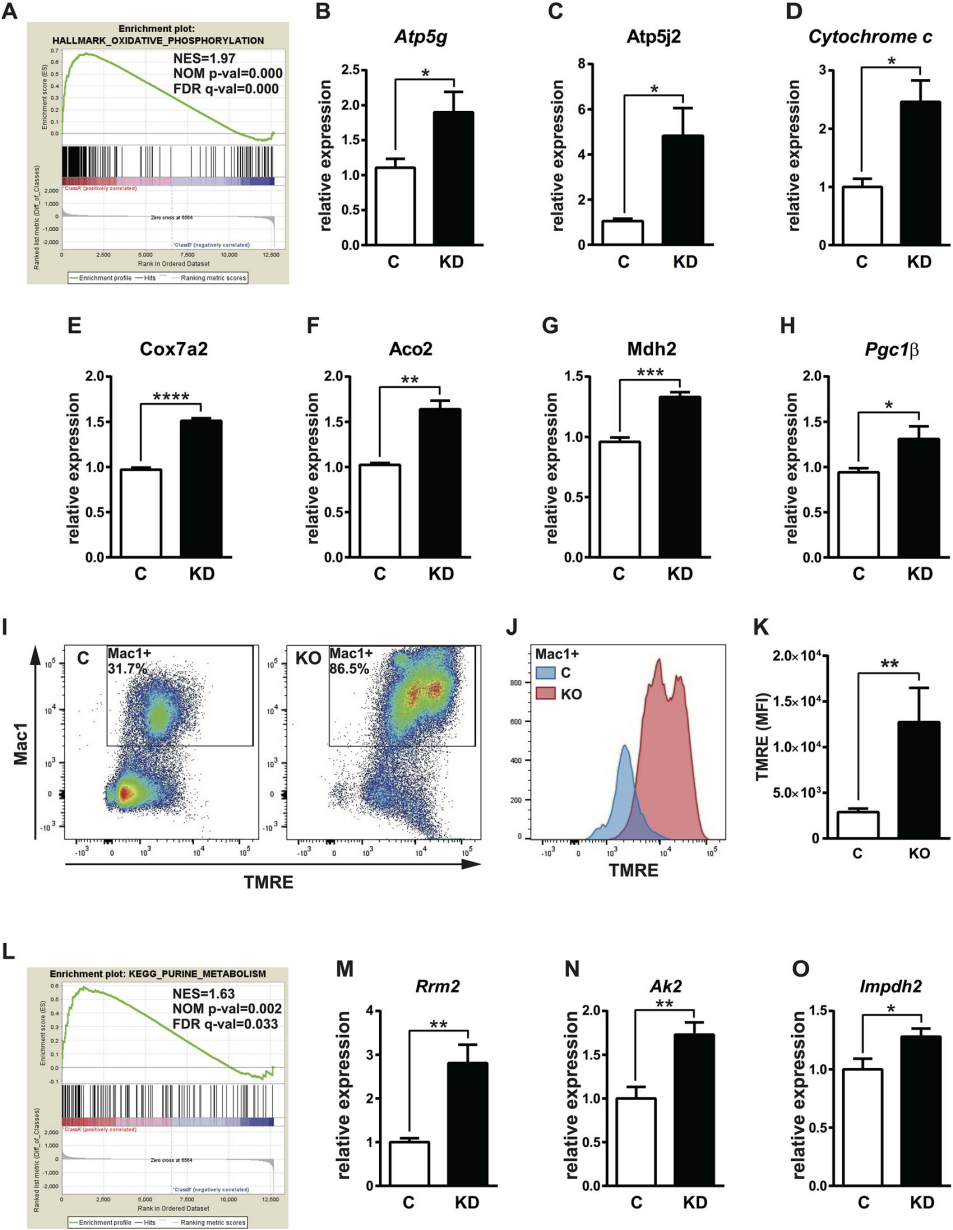
Significant metabolic changes in *Flcn*‐deficient osteoclast precursors. (*A*) Enrichment plots of gene set enrichment analysis (GSEA) (HALLMARK_OXIDATIVE_PHOSPHORYLATION: normalized enrichment score [NES] = 1.97, normalized *p* value [NOM p‐val] = 0.000, false discovery rate *q* value [FDR q‐val] = 0.000) Affymetrix GeneChip mediated comprehensive gene expression profiling was performed on RAW264.7 cells transfected with scramble siRNA or *Flcn* targeted siRNA. GSEA demonstrated that upregulated genes in *Flcn*‐deficient cells were significantly enriched in the oxidative phosphorylation gene set signature. (*B–G*) qRT‐PCR analysis of representative genes from the oxidative phosphorylation signature on RAW264.7 cells transfected with scramble siRNA (C) or *Flcn* targeted siRNA (KD). (*H*) The expression of *Pgc1β*, a master regulator for mitochondrial biogenesis in osteoclastogenesis, was significantly elevated in *Flcn* knockdown cells. Data represent means ± SD (triplicate, unpaired *t* test: *p < 0.05, **p < 0.01, ***p < 0.001, ****p < 0.0001). Representative data from three independent experiments are shown. (*I–K*) Mitochondrial membrane potential was measured by tetramethylrhodamine methyl ester (TMRM) staining of bone marrow cells from *Flcn* WT and KO mice at 14 days after pIpC injection. (*I*) Representative flow cytometry of Mac1 × TMRE. (*J*, *K*) Mean fluorescence intensity of TMRE in Mac1+ cells was measured and shown in a bar graph. Data are presented as mean with SD (*n* = 4 for WT and KO, unpaired *t* test: **p < 0.01). (*L*) Another significant enrichment plot by GSEA revealed significant positive correlation between upregulated genes in *Flcn*‐deficient RAW264.7 cells and the purine metabolism signature gene set. (KEGG_PURINE_METABOLISM: NES = 0.63, NOM p‐val = 0.002, FDR q‐val = 0.033). (*M–O*) qRT‐PCR analysis of representative genes from the purine metabolism signature on RAW264.7 cells transfected with scramble siRNA (C) or *Flcn* targeted siRNA (KD). (*M*) *Rrm2*, (*N*) *Ak2*, (*O*) *Impdh2*. Data represent means ± SD (triplicate, unpaired *t* test: *p < 0.05, **p < 0.01). Representative data from three independent experiments are shown.

For the purpose of elucidating the molecular mechanism of accelerated osteoclastogenesis in *Flcn*‐deficient cells, we performed comprehensive gene expression profiling. A gene set enrichment analysis (GSEA)^31^ demonstrated a highly significant gene enrichment for an oxidative phosphorylation signature in *Flcn* knockdown cells (Fig. 4 *A*, Supplemental Fig. S1*A*). As shown in Fig. 4 *A*, most genes involved in the oxidative phosphorylation gene set were upregulated in *Flcn*‐deficient cells compared with control cells. Gene expression of representative genes was confirmed by qRT‐PCR (Fig. 4 *B–G*). These results are consistent with our previous report that *Flcn* deficiency caused upregulation of *Pgc1α*, a master regulator for mitochondrial biogenesis, resulting in increased mitochondria mass, oxidative phosphorylation, and ATP production in striated muscle, heart, and kidney cancer cell lines.^15^, ^16^ As we have shown in Fig. 3 *H–K*, *Pgc1α* and *Pgc1β* expression is regulated by Tfe3. Indeed, *Pgc1β* expression was significantly higher in *Flcn* knockdown cells, suggesting that it has an important role in the dramatic shift of gene expression toward oxidative phosphorylation (Fig. 4 *H*). To see the physiological outcome of this characteristic gene expression profile, we measured mitochondrial membrane potential by TMRE staining on bone marrow–derived Mac1‐positive cells from *Flcnf/f* control mice and *Flcnf/f*, *Mx1‐Cre* mice at 2 weeks after pIpC injection. Indeed, the mitochondrial membrane potential was significantly higher in *Flcn* KO Mac1+ cells. (Fig. 4 *I–K*). In addition to oxidative phosphorylation, GSEA demonstrated significant gene enrichment for purine metabolism in *Flcn* knockdown RAW264.7 cells (Fig. 4 *L*, Supplemental Fig. S1*B*). Many genes listed in the purine metabolism gene set, including *Rrm2*, *Ak2*, and *Impdh2*, were upregulated in *Flcn*‐deficient cells (Fig. 4 *M–O*). These results strongly suggest the importance of metabolic regulation directed by *Flcn* in osteoclastogenesis.

### Regulation of nucleotide production and purinergic signaling loop by Flcn

**Figure 5:**
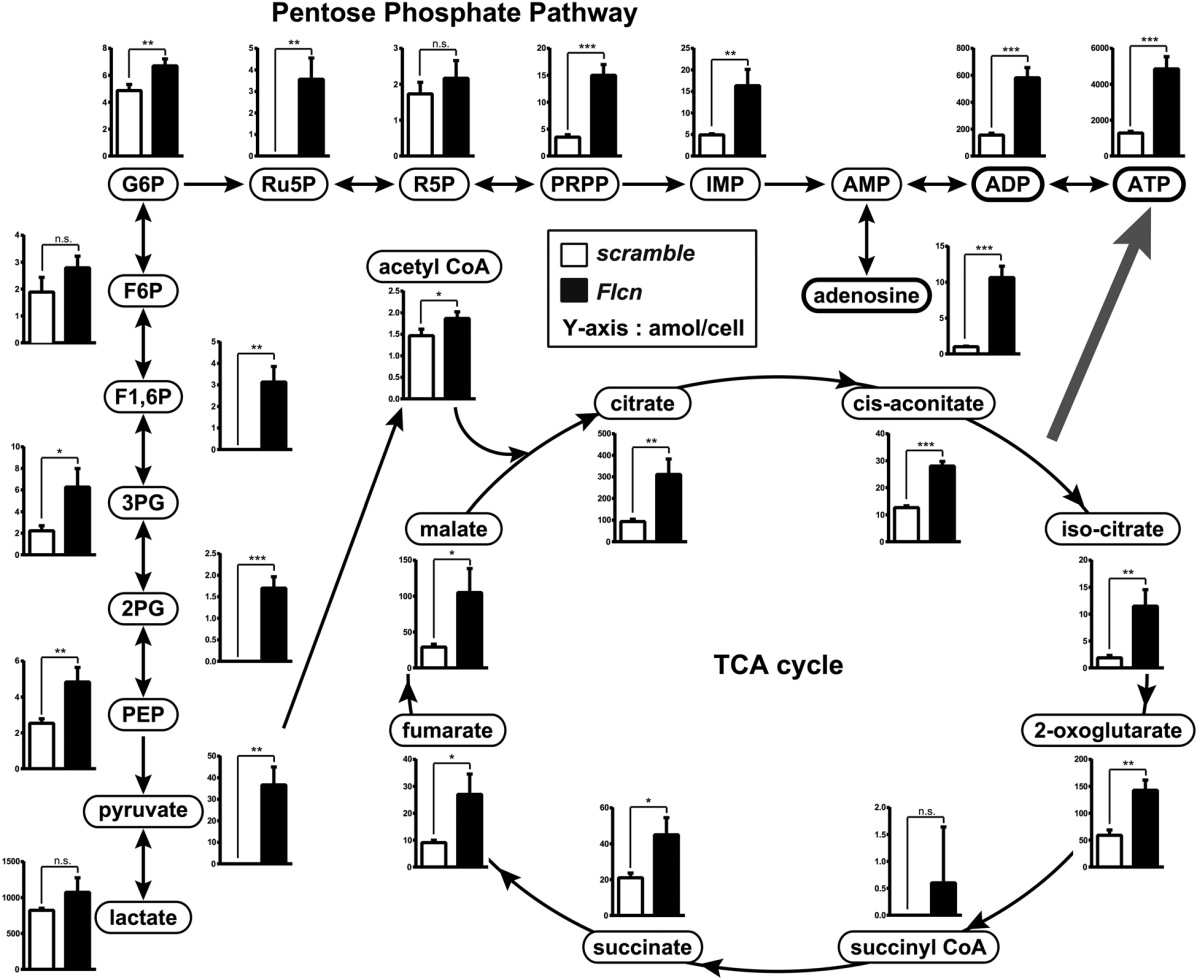
Upregulated nucleotide production and oxidative phosphorylation in *Flcn*‐deficient osteoclast precursors. Comprehensive metabolome analysis was performed on siRNA transfected Raw264.7 cells by CE‐TOF/MS. Cells were transfected with siRNA followed by RANKL stimulation (40 ng/mL) after 24 hours of transfection and harvested after an additional 48 hours of culture. Amount of each metabolite was adjusted by cell number. Open bars indicate data of scramble siRNA transfected cells and closed bars indicate *Flcn* targeted siRNA transfected cells. Y axis indicates amol/cell. Data represent means ± SD (*n* = 3, unpaired *t* test: *p < 0.05, **p < 0.01, ***p < 0.001, ****p < 0.0001). Metabolites, including PRPP, which are produced by pentose phosphate pathway, were elevated in *Flcn*‐deficient cells. Purine nucleotides and adenosine were dramatically elevated in *Flcn*‐deficient cells.

**Figure 6:**
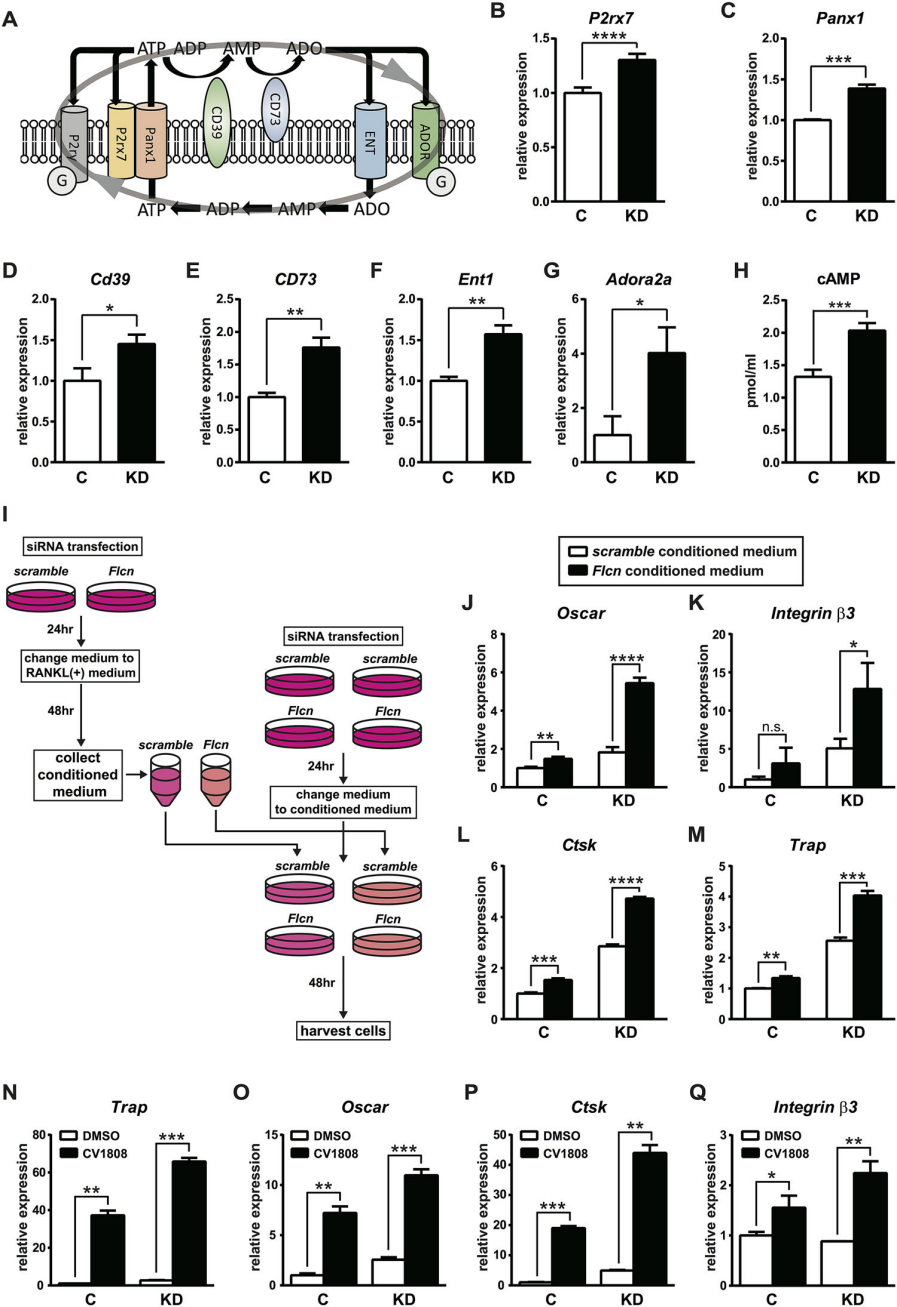
Regulation of nucleotide production and purinergic signaling loop by Flcn. (*A*) A scheme showing the purinergic signaling loop, which consists of controlled ATP release, extracellular metabolism of purine nucleotides by ectonucleotidases (CD39, CD73), stimulation of purinergic receptors by ATP and adenosine, and import of adenosine by ENT. (*B–G*) Gene expression was quantified by qRT‐PCR on siRNA transfected RAW264.7 cells cultured under the same condition as the metabolomics analysis in Fig. 5. The molecules involved in this purinergic signaling loop were expressed at significantly higher levels in *Flcn* knockdown cells. (*H*) The cellular cAMP level was quantified in siRNA transfected RAW264.7 cells, which were cultured under the same condition as *B–G*. (*I*) A scheme showing the conditioned medium experiment. (*J–M*) Conditioned medium from RAW264.7 cells transfected with scramble siRNA or *Flcn* targeted siRNA was collected. Then experimental RAW264.7 cells transfected with scramble siRNA or *Flcn* targeted siRNA were cultured with these conditioned medium for 48 hours, followed by qRT‐PCR analysis to quantify expression levels of osteoclastogenesis marker genes. (*N–Q*) RAW264.7 cells transfected with scramble siRNA or *Flcn* siRNA followed by 24‐hour incubation and additional 48‐hour incubation with 40 ng/mL of RANKL and DMSO or 50 μM of CV1808 (an Adora2 agonist). The expression levels of readout genes for osteoclastogenesis were quantified by qRT‐PCR. Data represent means ± SD (triplicate, unpaired *t* test: *p < 0.05, **p < 0.01, ***p < 0.001, ****p < 0.0001). Representative data from at least three independent experiments are shown.

To clarify the significance of this Flcn‐mediated metabolic regulation, we performed a metabolomics analysis on RAW264.7 cells by CE‐TOF MS.^25^ We compared quantitative metabolome profiling of *Flcn* knockdown and control RAW264.7 cells stimulated with RANKL at a concentration of 40 ng/mL for 48 hours. As we previously observed in skeletal muscle,^15^ loss of *Flcn* caused significant elevation of metabolites in the oxidative phosphorylation and the pentose phosphate pathways, which is represented by Ru5P and PRPP (Fig. 5). This metabolic change was accompanied by increased nucleotide production as observed by dramatic elevation of inosine monophosphate (IMP), from which adenine and guanine nucleotides are subsequently formed (Fig. 5). Among these purine metabolites, adenosine was prominently elevated in *Flcn* knockdown RANKL‐stimulated RAW264.7 cells (Fig. 5). Because adenosine, ADP, and ATP could work as signaling molecules to stimulate purinergic receptors (Fig. 6 *A*) and activate intracellular signaling pathways including cyclic AMP production, PI3K pathway, MAP kinase pathway, and calcium signaling,^32^, ^33^, ^34^, ^35^ we hypothesized that increased purinergic signaling had an essential role in enhanced osteoclastogenesis of *Flcn*‐deficient osteoclast precursors. The purinergic receptor P2rx7 is involved in controlled ATP release to the extracellular space and has an important role in physiological osteoclast differentiation.^36^, ^37^, ^38^, ^39^ *P2rx7* mRNA expression was significantly higher in RANKL‐stimulated *Flcn*‐deficient cells (Fig. 6 *B*). The hemichannel Pannexin1 (Panx1) is known to be responsible for P2rx7‐mediated controlled ATP release.^40^ *Panx1* mRNA expression was also significantly higher in *Flcn*‐deficient cells (Fig. 6 *C*). Released ATP is sequentially dephosphorylated by cell surface ectonucleotidases CD39 and CD73 and can stimulate adenosine receptors, which are G‐protein‐coupled receptors.^41^, ^42^, ^43^ Adenosine is transported across the plasma membrane through the nucleoside transporter ENT1 and utilized for nucleotide production (Fig. 6 *A*). Importantly, all of these players in the purinergic molecule–mediated signaling loop were expressed at significantly higher levels in *Flcn*‐deficient cells (Fig. 6 *B–F*). In addition, the expression of adenosine receptor *Adora2a* was also higher in *Flcn*‐deficient cells (Fig. 6 *G*). These results suggest a novel mechanism for regulation of the purinergic signaling loop by Flcn through increased nucleotide production and purine metabolism. Significant elevation of cAMP level in *Flcn*‐deficient RAW264.7 cells supports this idea (Fig. 6 *H*). To further investigate the role of a purinergic signaling loop for accelerated osteoclastogenesis in *Flcn‐*deficient cells, we cultured *Flcn* knockdown and control RAW264.7 cells with the conditioned medium collected from *Flcn* knockdown and control RAW264.7 cell culture (Fig. 6 *I*). As shown in Fig. 6 *J–M*, the conditioned medium from *Flcn*‐deficient cells enhanced osteoclastogenesis in *Flcn*‐intact cells. Furthermore, the enhancement of osteoclastogenesis by the conditioned medium from *Flcn*‐deficient cells was more significant in *Flcn* knockdown cells, (Fig. 6 *J–M*), which is consistent with the increased gene expression of purinergic signaling molecules in *Flcn*‐deficient cells (Fig. 6 *A–H*). Next, we stimulated *Flcn* knockdown and control RAW264.7 cells with an adenosine analog CV1808 and quantified the expression of readout genes for osteoclastogenesis. Consistent with the conditioned medium experiment, CV1808 increased the expression of osteoclastogenesis markers in *Flcn*‐intact cells as well as *Flcn*‐deficient cells (Fig. 6 *N–Q*). Importantly, *Flcn*‐deficient cells showed higher sensitivity to CV1808, which is also consistent with the increased gene expression of purinergic signaling molecules in *Flcn‐*deficient cells (Fig. 6 *A–H*).

### Flcn regulates osteoclastogenesis through a purinergic signaling loop

**Figure 7:**
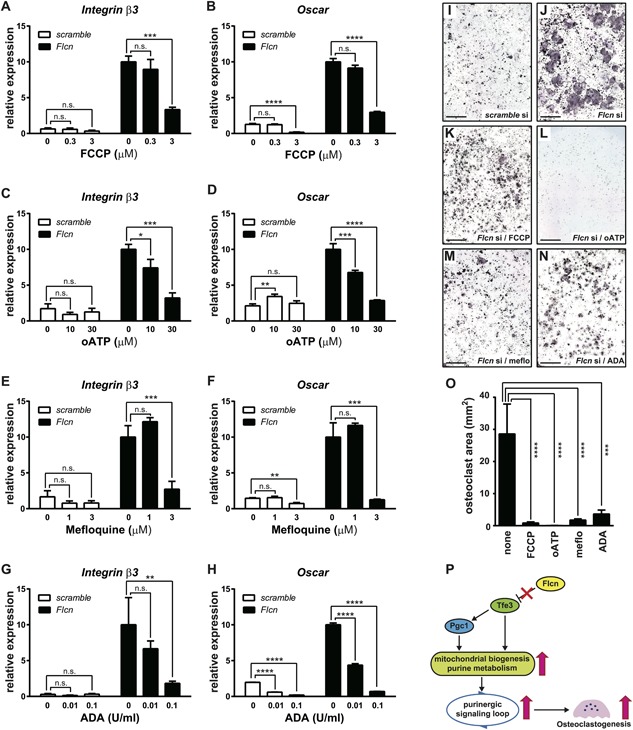
Flcn regulates osteoclastogenesis through a purinergic signaling loop. (A‐H) RAW264.7 cells transfected with scramble siRNA or *Flcn* siRNA followed by 24hr incubation and additional 48 hr incubation with RANKL stimulation at 40 ng/ml were treated with specific inhibitors for each molecule to block the purinergic signaling loop. The gene expression of readout genes for osteoclastogenesis, *Integrin β3* and *Oscar*, was quantified by qRT‐PCR to see the effect of purinergic signaling inhibitors on osteoclastogenesis. Cells were treated as follows. (*A*, *B*) FCCP to block mitochondrial ATP production; (*C*, *D*) oATP to inhibit P2rx7; (*E*, *F*) Mefloquine to block controlled ATP release by Panx1, which cooperates with P2rx7; (*G*, *H*) adenosine deaminase (ADA) to reduce extracellular adenosine. Data represent means ± SD (triplicate, *p < 0.05, **p < 0.01, ***p < 0.001, ****p < 0.0001). Representative data from at least three independent experiments are shown. (*I–N*) TRAP staining of RAW264.7 cells transfected with *Flcn* siRNA or scrambled siRNA followed by RANKL stimulation at 40 ng/mL for 96 hours with inhibitors of purinergic signaling loop. Osteoclastogenesis in *Flcn* knockdown RAW264.7 cells was significantly enhanced relative to controls (*I*, *J*, Fig. 2 *C*), and was inhibited by 3 μM of FCCP (*K*), 100 μM of oATP (*L*), 5 μM of Mefloquine (*M*), or 0.02 U/mL of ADA (*N*). Scale bars = 2 mm. (*O*) The area of multinucleated TRAP‐positive osteoclasts for each treatment is shown. Significant reduction of osteoclast area by the inhibitors for purinergic signaling loop was observed. Data represent means ± SD (triplicate, ***p < 0.001, ****p < 0.0001). Representative data from at least three independent experiments are shown. Scale bars = 2 mm. (*P*) A proposed model for metabolism‐mediated regulation of osteoclastogenesis by Flcn.

To further evaluate the role of this ATP‐mediated purinergic signaling loop in accelerated osteoclastogenesis under *Flcn* deficiency, we treated RANKL‐stimulated RAW264.7 cells with specific inhibitors for each of these molecules to block this purinergic signaling loop. Inhibition of ATP production by the mitochondrial uncoupler FCCP significantly suppressed *Integrin β3* and *Oscar* expression in *Flcn* knockdown cells (Fig. 7 *A*, *B*). The physiological significance of elevated intracellular ATP levels and increased *P2rx7* expression for osteoclastogenesis in *Flcn*‐deficient cells was evaluated by the P2rx7‐specific inhibitor oATP. Importantly, oATP treatment specifically suppressed expression of readout genes for osteoclast differentiation in *Flcn*‐deficient cells (Fig. 7 *C*, *D*). Panx1 is known to interact with P2rx7 and has a role in controlled ATP release to the extracellular space. Importantly, the Panx1 inhibitor Mefloquine significantly suppressed *Integrin β3* and *Oscar* expression in RANKL‐stimulated *Flcn*‐deficient cells (Fig. 7 *E*, *F*). Exported ATP is eventually converted to adenosine by Cd39 and Cd73 and can stimulate adenosine receptors.^41^, ^42^, ^43^ Addition of adenosine deaminase (ADA) to culture media catalyzes conversion of adenosine to inosine and reduces extracellular adenosine levels. In fact, ADA treatment significantly suppressed *Integrin β3* and *Oscar* expression in RANKL‐stimulated *Flcn*‐deficient cells (Fig. 7 *G*, *H*). Furthermore, we tested the effect of these drugs in an in vitro osteoclast differentiation assay. *Flcn* knockdown RAW264.7 cells differentiated into osteoclasts more effectively compared with control siRNA transfected cells (Fig. 7 *I*, *J*). However, osteoclast differentiation of *Flcn*‐deficient cells was significantly suppressed by treatment with FCCP, oATP, Mefloquine, and ADA (Fig. 7 *K–O*). These results strongly support the idea that Flcn modulates osteoclast differentiation through regulation of the purinergic signaling loop in osteoclast precursor cells.

## Discussion

In this work, we have demonstrated that Flcn has an essential role in the regulation of osteoclastogenesis. The induction of *Flcn* knockout in mouse bone marrow caused accelerated osteoclastogenesis, resulting in excess bone absorption and severe osteoporosis within 3 weeks. Omics analysis revealed dramatic metabolic changes in *Flcn*‐deficient pre‐osteoclasts with the activation of a purinergic signaling loop, which is responsible for accelerated osteoclastogenesis in *Flcn*‐deficient osteoclast precursors.

We and others have previously reported that Flcn regulates transcriptional activity of Tfe3 by controlling its subcellular localization.^20^, ^21^, ^28^ The elevated expression of *Integrin β3* in *Flcn*‐deficient osteoclast precursors was restored to near normal levels by additional *Tfe3* knockdown. Moreover, forced nuclear localization of Tfe3 itself induced *Pgc1α* and *Pgc1β* expression in *Flcn*‐intact cells. Furthermore, we confirmed that Tfe3 directly bound to the M‐box consensus sequences in promoter region of *Pgc1α* and *Pgc1β* by ChIP‐qPCR. These data underscore the importance of the Flcn‐Tfe3‐Pgc1 axis in the regulation of osteoclastogenesis through controlling metabolism. Indeed, comprehensive gene expression profiling revealed highly significant enrichment of oxidative phosphorylation genes in *Flcn*‐deficient osteoclast precursors. Osteoclasts are energy‐demanding cells and contain abundant mitochondria to produce ATP. During osteoclastogenesis, the metabolic state shifts toward a more oxidative state, accompanied by induction of *Pgc1β* expression and increased mitochondrial biogenesis.^1^, ^2^ Here, we have discovered an upstream regulatory signaling pathway for metabolic reprogramming that underscores the importance of the Flcn‐Tfe3‐Pgc1 axis for osteoclastogenesis.

Moreover, we found that genes involved in purine metabolism are significantly upregulated in *Flcn*‐deficient pre‐osteoclasts. qRT‐PCR showed upregulation of genes involved in nucleic acid metabolism, including *Rrm2*, *Ak2*, and *Impdh2*. Specifically, Impdh2 is the rate‐limiting enzyme for purine metabolism and has an essential role for HSC proliferation under stress conditions as a downstream effector of the p38‐MITF axis.^30^ Because Tfe3 belongs to the MITF family, it is reasonable that Impdh2 is upregulated in *Flcn*‐deficient cells through the activation of Tfe3. In fact, we confirmed *Impdh2* induction by forced Tfe3 nuclear localization (Fig. 3 *P*). These data further support the idea that the Flcn‐Tfe3 axis regulates purine metabolism toward osteoclastogenesis. The detailed mechanism of TFE3‐mediated regulation of purine metabolism awaits future investigation.

The unique alterations in gene expression profiling found in *Flcn*‐deficient cells were significantly reflected in the metabolome profiling. Dramatic elevation of purinergic metabolites including ATP, ADP, and adenosine was observed in *Flcn*‐deficient RANKL‐stimulated pre‐osteoclasts. This unique metabolome profile can be caused by the simultaneous activation of the pentose phosphate pathway and oxidative phosphorylation. These purinergic metabolites are continuously recycled by phosphorylation and dephosphorylation intracellularly and extracellularly. Controlled ATP release is mediated by the ATP receptor P2RX7 and a hemichannel PANX1.^44^ The released ATP can stimulate ATP receptors or is dephosphorylated to adenosine by cell surface ectonucleotidases, CD39 and CD73. Then, extracellular adenosine can stimulate adenosine receptors or be imported through transporters, ENTs, and CNTs.^36^, ^41^, ^42^, ^43^ This purinergic signaling loop can activate intracellular signaling pathways, including the PI3K pathway, MAPK pathway, and Ca2+ signaling. Indeed, the players involved in this purinergic signaling loop, *Panx1*, *P2rx7*, *Cd39*, *Cd73*, *Ent1*, and *Adora2a*, were all upregulated in *Flcn*‐deficient pre‐osteoclasts (Fig. 6 *B–G*). The blockage of this purinergic signaling loop by a variety of drugs targeting each molecule could efficiently inhibit accelerated osteoclastogenesis in *Flcn*‐deficient osteoclast precursors (Fig. 7 *A–O*). Taken together, loss of Flcn function alters the gene expression signatures of purine metabolism and oxidative phosphorylation through the Flcn‐Tfe3‐Pgc1 axis, leading to an accelerated purinergic signaling loop and enhanced osteoclastogenesis (Fig. 6 *P*).

In this study, we have shown that Flcn regulates osteoclastogenesis through a purinergic signaling loop. Many purinergic receptors may converge downstream through mutual signaling interactions.^34^, ^41^, ^43^ Identification of the critical purinergic receptors involved in the accelerated osteoclastogenesis under *Flcn* deficiency should be addressed in the future to characterize this newly identified Flcn role in purinergic regulation.

Although a critical role for purinergic signaling itself in osteoclastogenesis has been suggested,^45^, ^46^, ^47^ the upstream regulation mechanism for purinergic signaling in osteoclastogenesis has not been clarified. In this study, we have demonstrated a role for the Flcn‐Tfe3 axis in the upstream regulation of purinergic signaling in osteoclastogenesis. We have uncovered a novel molecular mechanism to control osteoclastogenesis through the metabolic reprogramming of oxidative phosphorylation and purine metabolism in the mouse system that could form the basis for development of innovative therapeutics for human diseases of abnormal bone metabolism. In addition, our new findings present a new paradigm of purinergic signaling in broader physiological events. Identification and clarification of the upstream signaling that regulates FLCN function and development of drugs to control FLCN function could be the next step toward the translational application of our findings.

## Disclosures

All authors state that they have no conflicts of interest.

## Supporting information

Supporting Data S1.

Supporting Figure S1.
