# Supplementary material for: Folliculin Regulates Osteoclastogenesis Through Metabolic Regulation
Source: J Bone Miner Res. 2018 Jun 26;33(10):1785–98. doi: 10.1002/jbmr.3477 (PMC6220829; doi:10.1002/jbmr.3477)
Supplement: Supplementary file 1 — Supporting Data S1. [file JBMR-33-1785-s001.docx]

**Supplemental Table 1 primer sequences for qRT-PCR**

| Gene |  | Sequence |
| --- | --- | --- |
| Aco2 | Forward | ccgatatgacctgctagagaagaa |
|  | Reverse | ggccgtaaacgcaggtatg |
| Adora2a | Forward | gtcctcacgcagagttccatc |
|  | Reverse | gaatgacagcacccagcaaa |
| Ak2 | Forward | gagattccgaagggcatcc |
|  | Reverse | cggtggccaaatgacaga |
| Atp5g | Forward | agttggtgtggctggatca |
|  | Reverse | gctgcttgagagatgggttc |
| Atp5j2 | Forward | tgccgctgaaggagaaga |
|  | Reverse | tccgaacgttgatgtacttgttg |
| Cathepsin K | Forward | atcttgtggactgtgtgactgagaatta |
|  | Reverse | gccgtggcgttatacatacaactt |
| Cd39 | Forward | ctggccaaggacattcagg |
|  | Reverse | tcacttacattcacaaccttctcgt |
| Cd73 | Forward | gcactctggttttgagatgga |
|  | Reverse | ctgtgtagagaaaggtgttggagtg |
| Cox5a | Forward | gggtcacacgagacagatga |
|  | Reverse | ggaaccagatcatagccaaca |
| Cox7a2 | Forward | accatcagcaccacttcacg |
|  | Reverse | accaagcgtcagagccatt |
| Cytochrome c | Forward | ggaggcaagcataagactgg |
|  | Reverse | tccatcagggtatcctctcc |
| Ent1 | Forward | cagttgtcattttggccatcc |
|  | Reverse | tcttccttttggctcctctcc |
| Flcn | Forward | gatgaccagtccctcagcaag |
|  | Reverse | ccaccacatccacagacagg |
| Gpnmb | Forward | gctacttcagagccaccatcacaa |
|  | Reverse | ggagatgatcgtacaggcttcca |
| Impdh2 | Forward | gacggcctcacctacaatga |
|  | Reverse | ggaaaccaatggggtctttagtg |
| Integrinb3 | Forward | aatgtcgtcagcctttaccagaa |
|  | Reverse | tcccgtaagcatcaacaatgag |
| Mdh2 | Forward | tcaacgtgcctgtcattgg |
|  | Reverse | gacttctgtgccagcctcct |
| Nfatc1 | Forward | ctcgaaagacagcactggagcat |
|  | Reverse | cggctgccttccgtctcatag |
| Oscar | Forward | ctggaactgctggtaacggat |
|  | Reverse | ccagggctgcacagagtcaata |
| P2rx7 | Forward | gcatagcagaggtgacggaga |
|  | Reverse | gcacttggccttctgacttg |
| Panx1 | Forward | cccaggagtgactgagaatgtg |
|  | Reverse | tactgctccacgattgggtactt |
| Pgc1α | Forward | ccgcaattctcccttgtatgtg |
|  | Reverse | ccgcttctcgtgctctttg |
| Pgc1β | Forward | cctcatgctggccttgtca |
|  | Reverse | tggcttgtatggaggtgtgg |
| Rrm2 | Forward | tgccatggaaaacatacactctg |
|  | Reverse | cagtcagccttcttcttcacaca |
| Tfe3 | Forward | gattggcgtgtctgctggt |
|  | Reverse | ggtggtagcgtgtagggttctc |
| Trap | Forward | gctggaaaccatgatcacct |
|  | Reverse | gagttgccacacagcatcac |

**Supplemental Table 2 primer sequences for ChIP-qPCR**

| Gene |  | Sequence |
| --- | --- | --- |
| Pgc1α | Forward | tccactctgacacacagcacac |
|  | Reverse | agtgacagcccagcctactttt |
| Pgc1β | Forward | ctgcaaagctcctggtatcaattac |
|  | Reverse | agcagaagcagtgcagtgaaag |

**Figure legends for Supplemental Figure 1**

**Supplemental Figure Significant metabolic changes in *Flcn* deficient osteoclast precursors.**

(A) Heat map of gene ranking within an oxidative phosphorylation signature (HALLMARK_OXIDATIVE_PHOSPHORYLATION). Red indicates higher expression and blue indicates lower expression in *Flcn* knockdown RAW264.7 cells. (B) Heat map of gene ranking within a purine metabolism signature (KEGG_PURINE_METABOLISM). Red indicates higher expression and blue indicates lower expression in *Flcn* knockdown RAW264.7 cells.
