# Supplementary figures and images for: Folliculin Regulates Osteoclastogenesis Through Metabolic Regulation
Source: J Bone Miner Res. 2018 Jun 26;33(10):1785–98. doi: 10.1002/jbmr.3477 (PMC6220829; doi:10.1002/jbmr.3477)

Supplemental Figure 1

A

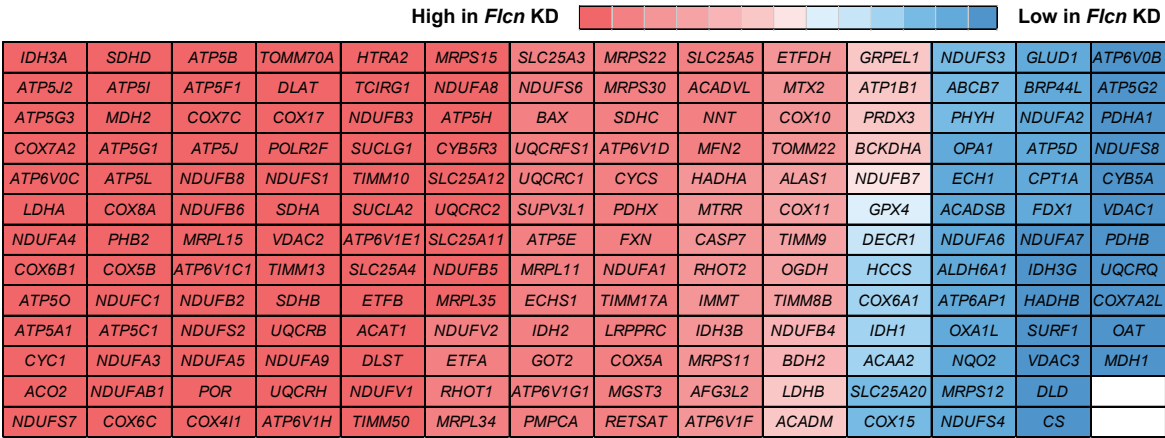

B

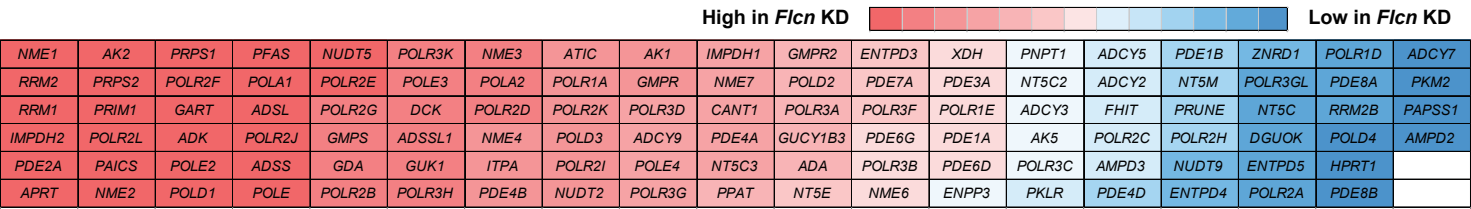

Supplement: Supplementary file 2 — Supporting Figure S1. [file JBMR-33-1785-s002.pdf]
